# Obstetric factors and neonatal outcomes of depressed skull fractures in newborns

**DOI:** 10.1007/s00404-024-07581-4

**Published:** 2024-06-13

**Authors:** Jihyun Choi, Iseop Cho, Tae Eun Kim, Hyeon Ji Kim, Jee Yoon Park, Chae-Yong Kim

**Affiliations:** 1 Department of Obstetrics and Gynecology, Seoul National University Bundang Hospital, Seoul National University College of Medicine, 82 Gumi-ro, 173 Beon-gil, Bundang-gu, Seongnam-si, Gyeonggi-do 13620 Republic of Korea; 2 Department of Neurosurgery, Seoul National University Bundang Hospital, Seoul National University College of Medicine, Seongnam, Republic of Korea

**Keywords:** Depressed skull fracture, Vacuum delivery, Birth injury, Neonate, Pediatric neurosurgery

## Abstract

**Purpose:**

To determine the obstetric factors affecting the development of depressed skull fracture in neonates.

**Materials and methods:**

This was a retrospectively cohort study on neonates born between July 2016 and August 2021. Neonates diagnosed with depressed skull fractures within one week of birth through X-ray and/or brain ultrasonography were included, and their mothers' obstetric characteristics were reviewed.

**Results:**

There were 12 cases in 6791 live births. Five women were over 35 years old. All except two were nulliparous. Five cases were delivered from labor induction and others presented with spontaneous labor. Except for two cases, delivery occurred within an hour after full cervical dilatation. Two cases were assisted by vacuum. None displayed fetal distress signs such as low Apgar scores below 7, meconium staining, and umbilical cord pH under 7.2. All depressed fractures were found in the right parietal area. Three cases resulted in focal hyperechoic lesion in brain ultrasonography and two of them showed small hemorrhage-like lesion in magnetic resonance imaging. All depressed skull fractures improved within 6 months in followed X-rays or ultrasonography.

**Conclusions:**

There was no definitely associated obstetric condition for depressed skull fracture of neonates although nulliparous women were majority of the affected cases.

## Introduction

Depressed skull fractures of neonates are defined as inward concavities of the calvarium and are called as “ping-pong fractures” because they resemble the smooth contour of the indented ball without definite evidence of frank break or disruption. The incidence of neonatal depressed skull fractures has been reported as approximately 1–2.5 per 10,000 live births, however varies according to the institutions or ethnicities [1]. During delivery and even in the whole pregnancy period, the neonatal calvaria protects the brain and, therefore, needs to withstand the pressure from various maternal structures including uterine wall, amniotic fluid, pelvic cavity, and vaginal canal for the cases with vagina delivery. Because neonatal skull bones are not fused yet, they tend to be depressed or dented inward instead of being broken into two or more pieces when the pressure increases beyond certain degree or produces the effective physical vector to the calvaria [2].

The etiology has not been identified clearly; however, birth trauma during delivery caused by instruments or other circumstances is likely suspected to result in depressed skull fractures of neonates [3, 4]. Nevertheless, there are a few reporting that defective molding secondary to normal maternal pelvic structures such as sacral promontory, lower uterin segment or uterine fibroids can also be the causes of neonatal depressed skull fractures since the fetal skull is characterized by lower mineralization and higher malleability than that of adults [1, 5]. The natural rotation of fetal position or prolonged pressure from the fetuses’ own body parts such as hand, foot, or body of other fetus in cases with multifetal pregnancy have been suggested as possible causes of neonatal depressed skull fractures [6].

Clinically, neonatal depressed skull fractures can be easily identified at delivery by the abnormal concavity or molding of the skull. Plan radiographs of the skull can be performed to diagnose the degree of deformation and cranial ultrasound is used to confirm the presence of intracerebral bleeding and hematomas [7, 8]. If the intracranial hemorrhage is not critically accompanied, the permanent or long-term adverse outcomes are known to be rare [3]. However, few cases have been reported to be complicated with epidural, subdural, and/or intracerebral hematomas, and because the growth rate of neonatal brain is relatively rapid, those hematomas have a chance to develop cerebral edema with brain compression or decreased cerebral blood flow that can result in brain dysfunction or epileptic seizures [9–11].

The treatment for the majority of depressed skull fractures without significant intracranial hemorrhage is usually close observation; nevertheless, interventions such as suction reduction or surgery have been suggested by surgeons for relatively severe cases although management guidelines are not clearly established [12]. The purpose of this study was to investigate the obstetric factors or characteristics related to the development of depressed skull fractures and to review the outcomes of affected cases from the single center experience.

## Materials and methods

This was a retrospective cohort study on live births over 24 weeks of gestation who had been delivered between July 2016 and August 2021. Neonates diagnosed as depressed skull fractures from X-ray and/or brain ultrasonography in one week after birth were included for the study population. The diagnosis of depressed skull fracture was defined as discontinuation of skull bone margin with depressed lesion in X-ray and/or brain ultrasonography either performed due to the clinical signs such as grossly depressed head or found incidentally at the evaluation for any indication. This study was approved by the Institutional Review Board (IRB).

Obstetric characteristics such as maternal age, parity, height, weight, the information on multifetal pregnancy, and other underlying conditions were reviewed through electronic medical records. Various factors affecting delivery including the presence of induction, epidural anesthesia, interval time from full dilatation of cervix to delivery, the use of vacuum assistance, and the application of episiotomy were evaluated to determine whether the circumstances of labor and delivery were complicated or not.

The characteristics of neonates such as gender, birthweight, head circumference, Apgar score at 1 min and 5 min, gross anomalies, umbilical cord blood gas analysis, the presence of meconium, and the admission to neonatal intensive care unit (NICU) were reviewed. The signs for the fetal distress during birth were defined as low Apgar scores, umbilical cord pH below 7.0, and the presence of meconium staining. Low Apgar scores were defined as 7 for both at 1 min and at 5 min. Detailed information about depressed skull fractures, the radiologic reports from X-ray, brain ultrasonography, and/or magnetic resonance imaging (MRI), and the latest follow-up evaluations on the skull lesions and neurodevelopment were collected as well.

## Results

There were 12 cases found to have depressed skull fractures in one week after birth among 6,791 live births (0.18%). The median maternal age was 34 years old (interquartile range 31–37) and gestational age at delivery was 37.0 (interquartile range 36–39). The median birthweight of the live births was 2,915 g (interquartile range 2435–3285). Male newborns were 48.2% (3272/6791). In the initial study population, approximately 80% was singleton pregnancy (5525/6791) and twins were 18% (1212 cases) and triplets were less than 1% (54 cases). The proportions of nulliparity and multiparity were 62.6% (4252/6791) and 37.4% (2539/6791), respectively. Vaginal delivery was performed in 43.0% (2923/6791) and among those, the rate of vacuum-assisted delivery was 9% (263/2,923). Among vaginal delivery, the proportions of nulliparity and multiparity were 60.2% (1,759/2,923) and 39.8% (1,164/2,923). Vacuum was applied in 13.6% (239/1,759) of nulliparous women and 2.1% (24/1,164) of multiparous women in those who had delivered vaginally.

Table 1 demonstrates maternal medical information of affected newborns. The median maternal age was 33 years old with the range of 30–42 years. About 5 out of 12 women were over 35 years old. Two cases were multiparous while all of the others were nulliparous. The median values of maternal height, weight, and body mass index were 165 cm (range 158–175 cm), 68.7 kg (range 59.8–97.3 kg), and 26.5 kg/m^2^ (20.2–34.8 kg/m^2^), respectively.

**Table 1:** Maternal obstetric characteristics of the study population

	Case 1	Case 2	Case 3	Case 4	Case 5	Case 6	Case 7	Case 8	Case 9	Case 10	Case 11	Case 12
Age (years)	36	33	31	33	42	42	30	32	38	33	33	42
Nulliparity	Yes	Yes	No	Yes	Yes	Yes	Yes	Yes	Yes	Yes	Yes	No
Height (cm)	167	175	158	161	165	163	165	159	168	160	166	167
Weight (kg)	59.8	61.8	60.4	80.4	76.6	70.2	65.4	67.2	77.8	61.1	76.2	97.3
BMI (kg/m^2^)	21.4	20.2	24.2	31.0	28.1	26.4	24.0	26.6	27.6	23.9	27.7	34.8
Number of fetuses	1	1	1	2 (twin A)	1	1	1	1	1	2 (twin A)	2 (twin A)	2 (twin A)
Maternal underlying conditions	Short cervix, PTL	PPROM	Previous precipitate delivery	DCDA twin, GDM	none	none	none	none	Previous conization	MCDA twin	DCDA twin	DCDA twin
Induction of labor	No	No	Yes	No	No	Yes	No	Yes	No	Yes	No	Yes
Epidural anesthesia	Yes	Yes	Yes	Yes	Yes	Yes	Yes	Yes	Yes	Yes	Yes	Yes
Interval from full dilatation of cervix to delivery (minutes)	145	42	8	15	19	43	39	140	26	31	35	22
Vacuum used	Yes	No	No	No	No	No	No	Yes	No	No	No	No
Episiotomy	Median	Median	No	Median	Median	Median	Median	RML	Median	Median	RML	Median

*BMI* body mass index, *PTL* preterm labor PPROM, preterm premature rupture of membranes, *DCDA* dichorionic diamniotic, *GDM* gestational diabetes, *MCDA* monochorionic diamniotic, *ROM* rupture of membranes, *RML* right mediolateral

There were four cases from twin pregnancies and the affected neonates were all twin A, the presenting or the first baby of twins. Case 1 was a high-risk pregnancy presented with preterm labor using tocolytics and short cervical length in mid-trimester and case 2 was admitted for preterm premature rupture of membranes. Case 3 had history of previous precipitate delivery and case 9 underwent cervical conization before the current pregnancy. Case 4 was diagnosed as gestational diabetes; however, it was well controlled during gestational period. Other cases did not have significant underlying diseases or obstetric complications.

Seven patients presented with spontaneous labor while five cases underwent induction of labor. All mothers of the study population received epidural anesthesia during labor according to the protocol that epidural anesthesia was initiated when active labor began. The median duration of the second stage of labor (from full dilatation of cervix to expulsion of fetus) was 33 min with the range of 8–145 min. Except for two cases (145 and 140 min for case 1 and case 8, respectively), most of women resulted in delivery in one hour from the full dilatation of cervix. Vacuum assisted delivery was performed in two cases (case 1 and 8). Episiotomy was done in all cases except for one case (case 3) and most of them (9/11) underwent median type of episiotomy.

Table 2 revealed neonatal outcomes including the detailed descriptions of depressed skull fractures. The median value of gestational age at delivery was 38.1 weeks (range 32.6–39.0 weeks) and preterm birth before 37 weeks of gestation was five cases. The affected newborns were composed of six male babies and six female babies. The median value of birthweight was 2940 g (range 1790–3330 g) and none of the neonates had abnormal birthweight percentile, neither below 10th percentile nor above 90th percentile. The median head circumference was 34.0 cm (range 32.9–39.0 cm); however, case 9 had small head circumference below 10th percentile of the gestational age. No newborn had adverse fetal distress signs such as low Apgar scores below 7 for both 1 min and 5 min or umbilical cord pH under 7.0. Meconium staining was not found in any of the study population. None of the affected newborns were admitted to NICU.

**Table 2:** Neonatal outcomes and the characteristics of depressed skull fractures from affected neonates

	Case 1	Case 2	Case 3	Case 4	Case 5	Case 6	Case 7	Case 8	Case 9	Case 10	Case 11	Case 12
Gestational age at delivery	38.1	32.6	38.6	33.6	38.9	38.7	39.0	38.6	38.1	36.1	35.7	36.0
Gender of neonate	Male	Male	Female	Male	Female	Female	Female	Male	Male	Female	Male	Female
Birthweight (grams)	3,230	1,960	2,965	1,790	3,330	2,660	2,920	3,115	2,960	2,385	2,820	2,995
… percentile	50–90	10–50	10–50	10–50	50–90	10–50	10–50	10–50	10–50	10–50	50–90	50–90
Head circumference (cm)	34.5	31.0	34.0	31.0	34.5	35.0	34.0	35.0	32.0	33.0	33.0	34
… percentile	50–90	50–90	50–90	10–50	50–90	10–50	10–50	50–90	3–10	50–90	50–90	50–79
Apgar score at 1 min	9	8	9	7	9	8	9	8	9	7	9	8
Apgar score at 5 min	10	10	9	9	10	9	9	9	10	9	10	10
Meconium staining	No	No	No	No	No	No	No	No	No	No	No	No
Congenital anomaly	No	Yes^a^	No	No	No	No	No	No	No	No	No	No
Cord pH	7.287	7.258	7.229	7.293	7.234	7.246	7.313	7.394	7.323	7.239	7.231	Not done
NICU admission	No	No	No	No	No	No	No	No	No	No	No	No
Location of lesion	Rt. Parietal	Rt. Parietal	Rt. Parietal	Rt. Parietal	Rt. Parietal	Rt. Parietal	Rt. Parietal	Rt. Parietal	Rt. Parietal	Rt. Parietal	Rt. Parietal	Rt. Parietal
Brain ultrasonography	Normal	Normal	Normal	PVE, mild	Focal hyperechoic lesion at Rt. high frontal subcortical region adjacent depressed fracture	Suspicious for focal WMI at Rt. Parietal area	PVE, mild	normal	normal	Focal hyperechogenic lesion at right parietal or temporal sulci or subcortical white matter	Normal	Normal
Brain MRI	Not done	Not done	Not done	Not done	Normal	Several dot-like T1 high/T2 low lesions, Rt parietal	Not done	Multifocal T2 high signal lesions in the both fronto-parietal deep WM	Not done	Subarachnoid hemorrhage along the right frontal and temporal and left occipital sulci	Not done	Not done
Age of imaging showing improved skull fracture (months)	6	6	6	2	4	6	3	4	4	4	3	6
Latest result of neurodevelopment	Normal	Normal	Normal	Normal	Normal	Normal	Normal	Delayed development	Normal	Normal	Normal	Normal
Age of last visit (months)	24	24	4	15	8	25	10	43	37	34	12	6

^a^Imperforate anus and bilateral hydronephrosis due to ureteropelvic junction obstruction

*NICU* neonatal intensive care unit, *Rt*. right, *PVE* periventricular echogenicity, *WMI* white matter injury, *MRI* magnetic resonance imaging

**Fig. 1:**
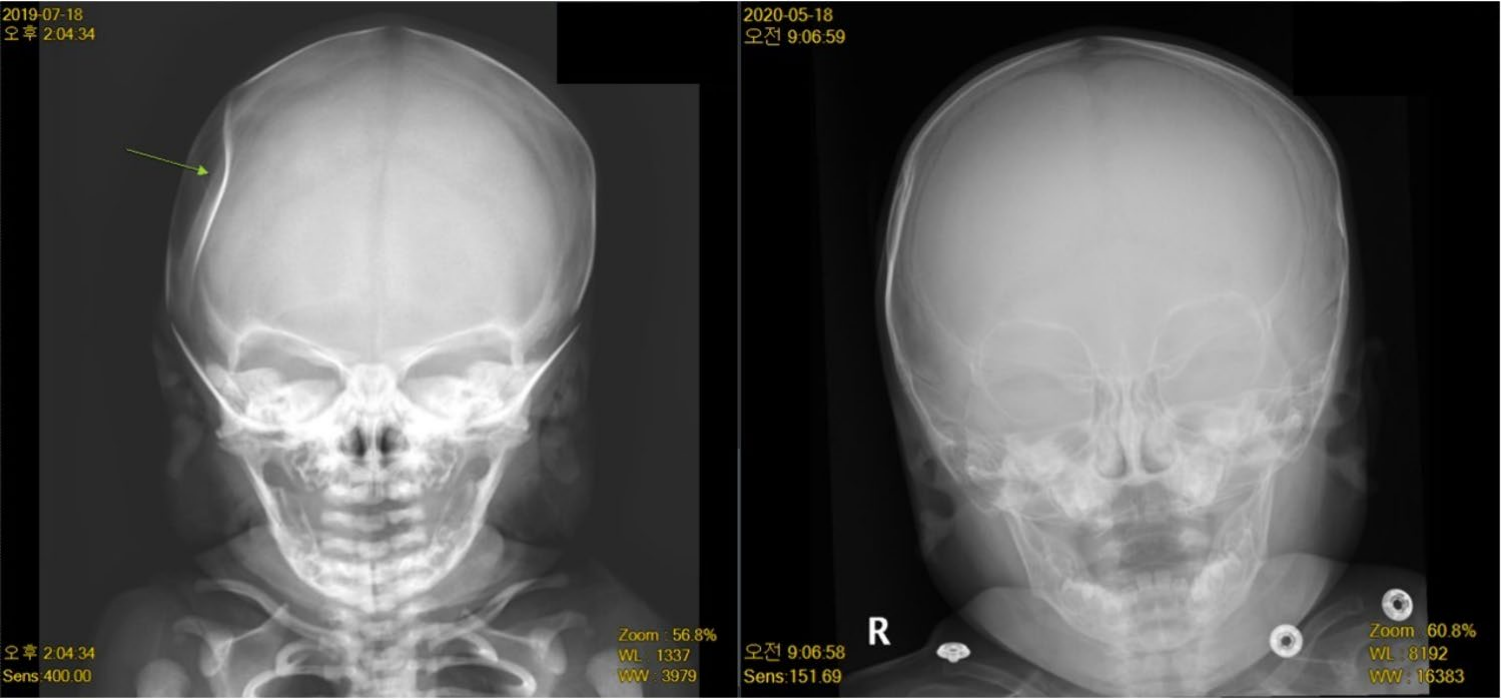
The skull X-ray images at the birth and at the latest visit of case 7 (left, depression fracture at the right parietal bone taken on day 1 after birth—July 18, 2019; right, interval improved state of right parietal area depressed fracture taken on when she became 10 months old—May 18, 2020). She was born through spontaneous vaginal delivery at gestational age of 39+0 weeks and her birthweight was 2,920 g (10–50 percentile)

All neonates underwent initial neurologic examinations including primitive reflexes, however there was no case showing abnormal signs. All depressed fractures were found in the right parietal area. Brain ultrasonography was performed for all the neonates and seven cases had normal results. Except for case 8, only 2 out of 12 (17%) patients had intracranial lesions along with DSF, which were resolved through observation. Cases 4 and 7 showed mild periventricular echogenicity (PVE) and Cases 5, 6, and 10 had brain sonography reporting focal hyperechoic lesions, which could be associated with white matter injury. All of three cases underwent MRI for further evaluation and two of them revealed the possibility of small hemorrhage-like lesion. All of the depressed skull fractures were improved at the followed examinations either X-rays or ultrasonography and as well as the gross inspections (Fig. 1). The median time at the test showing improvement was 4 months (range 2–6 months).

Case 8 was diagnosed with global developmental delay at 19 months. His skull X-ray had shown improvement at 4 months after the initial diagnosis; however, MRI was followed after approximately 2 years later to exclude any brain lesion that might affect global developmental delay. Multifocal T2 high signal lesions in both fronto-parietal deep white matter, suspicious for either ischemic lesions or sequelae of a congenital infection, were observed and chromosomal microarray analysis (CMA) reported duplication of 5p13.1 (2.6 Mb) and duplication of 5q23.1 (987 kb), which have been known as variant of unknown significance. This child has been currently diagnosed as autism spectrum disorder and followed up in neuropsychiatric department. Except for case 8, all of the neonates demonstrated normal neurodevelopment without seizures, involuntary movements, developmental delay, or cerebral palsy at the latest follow-up visits. The median age at the most current evaluation was 19 months with range from 4 to 43 months. All of the neonates were continuously observed by both neonatology and neurosurgery department, nevertheless none of them received interventions such as reduction with suction or surgery.

## Discussion

The principle implication of this study was that there was no significantly associated obstetric risk factor found in the group of newborns affected with depressed skull fractures, except for parity. Most of the mothers were nulliparous. In fact, nulliparous women tend to have more difficult or longer labor compared to multiparous ones. We investigated the interval time from full dilatation of cervix to delivery and the median duration was 33 min with relatively wide range from 8 to 145 min. The median duration of the second stage of labor, which is defined as the interval time from the fully dilated cervix to delivery, has been known as approximately 50 min for nulliparous women and 20 min for multiparous women [13, 14]. It took less than 30 min for two multiparous women from full dilatation of the cervix to delivery, while two of 10 (20%) nulliparous women had longer duration of the second stage of labor than the known research or guidelines.

The use of vacuum assistance, which is the well-known risk factor for neonatal depressed skull fractures, was found only in those above two cases with relatively longer duration of the second stage of labor. Since the incidence of abnormally prolonged labor was not remarkably high in the affected cases, it was hard to confirm the positive association between the complicated labor and the development of neonatal depressed skull fractures. All the mothers received epidural anesthesia and four cases underwent induction of labor. Among the twelve affected cases, there were five women of maternal age over 35 years. Maternal somatotypes were not consistent and the underlying conditions were various. Preterm birth before 37 weeks of gestation was five cases; however, all of the neonates were born after 32 weeks of gestation, which means none was from the early preterm birth. While selecting the affected neonates from the total of live births in the institution, none was found in preterm birth newborns before 32 weeks of gestation.

The birthweight of the affected neonates were from 1790 g to 3330 g, and none of them were too small or too big according to the percentile decided by gestational age. Small babies, usually defined as below 10th percentile, might have higher chance of lower mineralization in bones and large babies, defined as above 90th percentile, might have higher risk of complicated labor and delivery. Nevertheless, none of the affected newborns were identified as small-for-gestational age or large-for-gestational age. The clinical signs of fetal distress during birth defined as low Apgar scores below 7, low umbilical cord pH below 7.0, and the presence of meconium staining or admission to NICU were not found in the affected neonates. Therefore, complicated labor, which might result in adverse neonatal outcomes, was not evident in the population composed of affected newborns. In other words, the neonatal depressed skull fractures did not seem to be directly associated with adverse perinatal outcomes.

Observing the details of skull lesion, all the location was right parietal bone. This could be explained by the cardinal movement, the rotation of fetal body position to adapt the cavity in birth canal. The cardinal movements mostly occur during the second stage of labor and accordingly disproportion or resistance of the maternal pelvic cavity and the fetus becomes apparent [15]. In general, the vertex or cephalic presentation of fetuses enter the pelvis with the sagittal suture lying in the transverse diameter of pelvis [16]. When fetal head engages into the pelvic cavity, occiput usually comes in the left side, resulting in the right parietal bone exposed to the anterior pelvic bones, which are narrower than the posterior structures [17, 18]. In this point of view, one can assume the inappropriate engagement of fetal head to pelvic cavity or birth canal has a chance to spontaneously make depressed skull fracture even without trauma from outside of maternal body or instruments of medical personnel.

Although the limitations from sample size and from the descriptive study design based on observation of retrospective cohort are definitely weak points of the study, we have to admit that the incidence of neonatal depressed skull fractures is too rare to design any comparison to controls. The study was performed based on a sample group with a high proportion of high-risk pregnancies. In addition, we have not used forceps for delivery assistance over a decade. Here we found the prevalence of neonatal skull depressed fracture in our institution as the proportion of 0.18%. The low incidence of neonatal depressed fracture makes it hard to identify the risk factors or preventative methods. In fact, a large sample sized trial or prospective study in the published literature have not been available, but mostly case reports from the very different situations, which make it difficult to draw a conclusion for proper management plans (Table 3). Therefore, the information on the etiology and appropriate treatment based on the long-term prognosis are lacking yet.

**Table 3:** Literature review on neonatal depressed skull fractures (DSF

References	Number of cases	Inclusion criteria or details of cases	Location of DSF	Presence of intracranial hemorrhage	Management	Prognosis	Implications of the research
Loeser et al. [23]	3	(1) 3600 g male, forceps delivery (2) 2647 g female born at 36 weeks, forceps delivery (3) 3960 g female, forceps delivery	(1) Right parietal (2) Right parietal (3) Right frontal	Blood in CSF in case 2	Observation only	None of the children had seizures or neurological deficit	Surgical elevation is not mostly necessary for DSF caused by birth trauma Factors to reduce the need for surgical elevation are: 1) minimal depression less than 2 cm, 2) depression over the major venous sinus without signs of increased intracranial pressure or cerebral dysfunction
Saunders et al. [24]	3	(1) 980 g male born at 28 weeks, breech extraction vaginal delivery (2) 3200 g male, cesarean section (3) 3430 g male born at 38 weeks, cesarean section	1) Right frontal 2) Left parietal 3) Left frontal	None	1), 2) Obstetrical vacuum extractor was used to elevate DSF 3) Obstetrical vacuum extractor using small transparent breast shield as suction cup was used	-DSF elevated successfully without complications -Infants were discharged well	Obstetrical vacuum extractor method can safely elevate DSF without neurological signs
Roman Garza-Mercado et al. [5]	3	(1) 2600 g male, breech vaginal delivery (2) 2200 g male born at 35 weeks, cesarean section (3) 3700 g female born at term gestation, cesarean section	(1) Left parieto-occipital (2) Right temporo-parietal 3) Right parieto-occipital	(1) Blood in CSF (2) None (3) None	Neurosurgical elevation (*n* = 3)	(1) Seizure developed and controlled at discharge with improved general condition and slow psychomotor development was observed 2, 3) Normal neurological examination postoperatively	DSF can be found even in uncomplicated spontaneous delivery or cesarean section
Eisenberg et al. [25]	1	3700 g female born at 37 weeks, cesarean section	Right parietal	None	Observation only	CR at 6 months of age	DSF may resolve spontaneously, therefore if complications such as neurologic signs or signs of intracranial abnormalities are not present, surgical intervention is not required
Abbassioun et al. [26]	10	Ten neonates with non-iatrogenic intrauterine skull fractures	Not described	Two cases: none Eight cases: not described	Spontaneous elevation (*n* = 1) Vacuum extraction (*n* = 4) -Neurosurgical elevation (*n* = 5)	Not described	Non-operative management is considered the first option for DSF without intracranial complication, rather than operations -Neurosurgical intervention is the treatment of choice when intracranial lesion is found at the imaging study
Dupuis et al. [2]	68 Spontaneous delivery (n = 18) Instrumental delivery (n = 50)	Newborns with DSF between 1990 and 2000	Not described	Only in instrumental delivery group -Epidural or subdural hematoma (*n* = 14) -Parenchyma lesion (*n* = 7) -At least 1 intracranial-associated lesion (*n* = 15)	Cases required neurosurgery -Spontaneous group (89%) -Instrumental group (86%)	Only in instrument group -Persistent severe motor disability (*n* = 2) -Esthetic sequels (*n* = 7)	Intracranial lesions were significantly more frequent in the instrumental group than spontaneous group (30% vs 0%, *P* = 0.02)
Hung et al. [27]	25 -Minor depression (*n* = 11) -Larger depression (*n* = 14)	Newborns with DSF between 1985 and 2001	Not described	None	-For minor DSF (< 5 mm in depth), conservative management only -For larger DSF (> 5 mm in depth and usually > 2 cm in length), vacuum extraction was used	-In minor depression, DSF resolved spontaneously in eight cases within 1–6 months -In larger depression, all resolved after observation except for one (complete recovery after vacuum use) -No neurological deficit or later epilepsy was noted	Non-surgical management can be the treatment of choice for infants with simple DSF, whereas vacuum extraction is an option for larger and deeper depressions to obtain prompt resolution without taking additional risk
Hanlon et al. [28]	1	3460 g male born at term gestation, vacuum extraction delivery	Right parietal	None	Observation only	CR at 4 months of age	Surgical or vacuum elevation treatment of DSF should be reserved for the adverse event or lack of spontaneous resolution within six months
Dharmaraj et al. [8]	1	Female born at term gestation, cesarean section	Right parietal	None	Neurosurgical elevation	Neurological examination was normal, no residual neurological deficit	-Persistent disabilities are rare for DSF -Instrumental deliveries are more likely to be associated with intracranial lesions such as subdural hematomas than spontaneous vaginal deliveries
Basaldella et al. [1]	2	(1) 2780 g female born at 38 weeks, cesarean section (2) 3660 g female born at 36 weeks, cesarean section	1) Left parietal 2) Right parietal	None	Observation only	Both CR at 8 months of age	Even without complicated vaginal delivery or history of external trauma, DSF can occur spontaneously
Flannigan et al. [29]	1	Male born at 34 weeks 4 days, cesarean section	Right parietal	None	Observation only	CR at 3 months of age	Spontaneous resolution of congenital DSF from faulty fetal packing often occurs over the first few months without cosmetic or neurodevelopmental sequelae
Cizmeci et al. [30]	1	3240 g baby born at 38 weeks, vaginal delivery	Right frontal (5 × 4 cm sized)	None	Surgical elevation	No cosmetic or neurological complication at 1 month of age	There is insufficient evidence to support the specific treatment option and timing of correction for DSF
Preston et al. [3]	1	3240 g female born at 38 weeks 6 days, cesarean section	Right temporo-parietal	None	Observation only	Reduction in size of depression with bony remodeling and otherwise normal skull growth development was observed	DSF is associated with forceps delivery both in vaginal delivery and in cesarean section, however are much rarer in deliveries without instrumentation
Loire et al. [31]	1	Female born at 38 weeks, cesarean section	Left parietal	None	Not described	Not described	DSF has good prognosis if the newborn presents with normal neurological examination at birth
Ilhan et al. [32]	1	3250 g male born at 39 weeks, cesarean section	Right parietal	None	Observation only	CR at 6 months of age	Most untreated ping-pong fractures resolve spontaneously within 6 months
Ballestero et al. 33	1	4420 g male born at 37 weeks 4 days, cesarean section	Right fronto-temporal	None	After 72 h of observation, the suction cup vacuum method was applied	CR after elevation by suction cup vacuum method	Suction cup vacuum method is a feasible method to reduced DSF in children and is associated with minimum complications

*DSF* depressed skull fractures, *CSF* cerebrospinal fluid, *CR* complete resolution

Dupuis et al. demonstrated a retrospective study of 68 neonates with depressed skull fractures from a cohort of ten years [2]. They compared the spontaneously affected cases (*n* = 18) to those caused by forceps-assisted deliveries (*n* = 50) and reported the rate of epidural or subdural hematoma was significantly higher in forceps delivery group than spontaneous group (28% vs. 0%, *p* = 0.029). This study was performed with a relatively large sample size considering the low incidence of neonatal depressed skull fractures; however, forceps delivery has been known to be associated with higher maternal and perinatal morbidity than vacuum-assisted delivery, the other tool for the instrumental delivery [19, 20]. Thus, physicians tend to use vacuum more rather than forceps for instrumental delivery although the choice is generally based on personalized experience of the attending physician [21, 22]. In fact, Dupuis et al. reported two cases with persistent and severe motor disabilities among those affected cases who had been delivered by assistance of forceps. In the study population of current study, cases with forceps delivery were not included since we perform vacuum delivery only.

According to this study, the depressed fracture for newborns did not seem to have specific risk factors except for nulliparity since there was only one multiparous woman in the cases analyzed. The progression of labor or time spent until delivery could not be defined “prolongated” in most of the cases. In addition, most of the neonates were not excessively large and some were relatively small for their gestational age. Therefore, certain circumstance of abnormal or difficult labor cannot be described to be associated with neonatal skull depressed fractures. The prognosis of the affected neonates was relatively optimistic. All of the neonates revealed improvement of the skull fracture in shape followed later on in six months and demonstrated normal neurodevelopment until the latest visits although long-term observation is necessary and still ahead. None of the newborns received surgical intervention or reduction by suction. Therefore, we agree to several previous researchers who had suggested that close observation without surgical management is likely to be recommended when critical intracranial complications such as hemorrhage are not definitely suspected ) (Table 3).

Research on neonatal depressed skull fracture is essential to understand the risk factors and to establish delivery protocols to prevent it. Moreover, the medico-legal issues based on the suspicion to association between delivery procedures and perinatal adverse outcomes have been widely experienced by numerous obstetricians and neonatologists. If the definite risk factor for depressed skull fracture cannot be pointed out through accumulation of reliable evidence, one should not blame attending physician with indirect assumption of the cause-and-effect relationship between birth process and the skull lesions. Therefore, sharing affected cases and further systematic studies on immediate newborn injuries or abnormalities are significantly important to protect physicians in clinical practice as well as to counsel the parents of affected newborns.

## Conclusions

There was no definitely associated obstetric factor for the development of depressed skull fracture in neonates although nulliparous women were majority of the affected cases. The neonatal outcomes followed up until approximately one year from birth did not show any sequelae without surgical interventions.

## Data Availability

The datasets generated during and/or analyzed during the current study are available from the corresponding author on reasonable request. All data generated or analyzed during this study are included in this published article.

## References

[1] Basaldella L, Marton E, Bekelis K, Longatti P (2011). Spontaneous resolution of atraumatic intrauterine ping-pong fractures in newborns delivered by cesarean section. J Child Neurol.

[2] Dupuis O, Silveira R, Dupont C, Mottolese C, Kahn P, Dittmar A (2005). Comparison of “instrument-associated” and “spontaneous” obstetric depressed skull fractures in a cohort of 68 neonates. Am J Obstet Gynecol.

[3] Preston D, Jackson S, Gandhi S (2015). Non-traumatic depressed skull fracture in a neonate or ‘ping pong’ fracture. BMJ Case Rep.

[4] Kong CW, To WWK (2024). Precision of vacuum cup placement and its association with subgaleal hemorrhage and associated morbidity in term neonates. Arch Gynecol Obstet.

[5] Garza-Mercado R (1982). Intrauterine depressed skull fractures of the newborn. Neurosurgery.

[6] Cooke RWI, Fanaroff AA, Martin RJ (1993). Neonatal—perinatal medicine: diseases of the fetus and infant 5th ed. Pediatric Pulmonology.

[7] Miller JD, Jennett WB (1968). Complications of depressed skull fracture. Lancet.

[8] Dharmaraj ST, Embleton ND, Jenkins A, Jones G (2009). Depressed skull fracture in a newborn baby. Arch Dis Child Fetal Neonatal Ed.

[9] Raynor R, Parsa M (1968). Nonsurgical elevation of depressed skull facture in an infant. J Pediatr.

[10] Natelson SE, Sayers MP (1973). The fate of children sustaining severe head trauma during birth. Pediatrics.

[11] Ross G (1975). Spontaneous elevation of a depressed skull fracture in an infant. Case report J Neurosurg.

[12] Zalatimo O, Ranasinghe M, Dias M, Iantosca M (2012). Treatment of depressed skull fractures in neonates using percutaneous microscrew elevation. J Neurosurg Pediatr.

[13] Laughon SK, Branch DW, Beaver J, Zhang J (2012). Changes in labor patterns over 50 years. Am J Obstet Gynecol.

[14] Kilpatrick SJ, Laros RK (1989). Characteristics of normal labor. Obstet Gynecol.

[15] Iversen JK, Kahrs BH, Eggebo TM (2021). There are 4, not 7, cardinal movements in labor. Am J Obstet Gynecol MFM.

[16] Iversen JK, Jacobsen AF, Mikkelsen TF, Eggebo TM (2021). Structured clinical examinations in labor: rekindling the craft of obstetrics. J Matern Fetal Neonatal Med.

[17] Wittman AB, Wall LL (2007). The evolutionary origins of obstructed labor: bipedalism, encephalization, and the human obstetric dilemma. Obstet Gynecol Surv.

[18] Katzir T, Brezinov Y, Khairish E, Hadad S, Vaisbuch E, Levy R (2023). Intrapartum ultrasound use in clinical practice as a predictor of delivery mode during prolonged second stage of labor. Arch Gynecol Obstet.

[19] Seki H (2018). Complications with vacuum delivery from a forceps-delivery perspective: Progress toward safe vacuum delivery. J Obstet Gynaecol Res.

[20] Muraca GM, Sabr Y, Lisonkova S, Skoll A, Brant R, Cundiff GW (2019). Morbidity and mortality associated with forceps and vacuum delivery at outlet, low, and midpelvic station. J Obstet Gynaecol Can.

[21] Gnanasekaran V, Kanamma S, Dhinakaran S, Kalaiselvi J (2021). Assisted vaginal delivery-preference of vacuum or forceps among obstetricians. J Pharma Res Int.

[22] Caudwell Hall J, Shek C, Langer S, Dietz HP (2020). The effect of replacing vacuum with forceps in operative vaginal delivery: an observational study. Int Urogynecol J.

[23] Loeser JD, Kilburn HL, Jolley T (1976). Management of depressed skull fracture in the newborn. J Neurosurg.

[24] Saunders BS, Lazoritz S, McArtor RD, Marshall P, Bason WM (1979). Depressed skull fracture in the neonate: report of three cases. J Neurosurg.

[25] Eisenberg D, Kirchner S, Perrin E (1984). Neonatal skull depression unassociated with birth trauma. Am J Roentgenol and Radium Ther.

[26] Abbassioun K, Amirjamshidi A, Rahimizadeh A (1986). Spontaneous intrauterine depressed skull fractures. Child’s Nerv Syst.

[27] Hung K-L, Liao H-T, Huang J-S (2005). Rational management of simple depressed skull fractures in infants. J Neurosurg Pediatr.

[28] Hanlon L, Hogan B, Corcoran D, Ryan S (2006). Congenital depression of the neonatal skull a self limiting condition. Archives Dis Child-Fetal Neonatal Edit.

[29] Flannigan C, O'Neill C (2011). Faulty fetal packing. Case Rep.

[30] Cizmeci MN, Kanburoglu MK, Cemil B, Gokce EC, Tatli MM (2014). Ping pong fracture in the newborn: illustration of a case. Acta Neurol Belg.

[31] Loire M, Barat M, Kinkembo LM, Lenhardt F, M’buila C (2016). Spontaneous ping-pong parietal fracture in a newborn. Arch Dis Child Fetal Neonatal Ed.

[32] Ilhan O, Bor M, Yukkaldiran P (2018). Spontaneous resolution of a ‘ping-pong’fracture at birth. Case Rep.

[33] Ballestero MF, De Oliveira RS (2019). Closed depressed skull fracture in childhood reduced with suction cup vacuum method: case report and a systematic literature review. Cureus.

